# Osteoprotegerin (OPG) protects ovarian cancer cells from TRAIL-induced apoptosis but does not contribute to malignant ascites-mediated attenuation of TRAIL-induced apoptosis

**DOI:** 10.1186/1757-2215-5-34

**Published:** 2012-11-15

**Authors:** Denis Lane, Isabelle Matte, Claudine Rancourt, Alain Piché

**Affiliations:** 1 Département de Microbiologie et Infectiologie, Université de Sherbrooke, 3001 12ième Avenue Nord, Sherbrooke, J1H 5N4, Canada

**Keywords:** Osteoprotegerin, TRAIL, Ovarian carcinoma, Resistance, Ascites, Apoptosis

## Abstract

**Background:**

Resistance to apoptosis is a major problem in ovarian cancer and correlates with poor prognosis. Osteoprotegerin (OPG) is a secreted factor in malignant ascites and acts as a decoy receptor for receptor activator of NF-κB ligand (RANKL) and tumor necrosis factor-related apoptosis-inducing ligand (TRAIL). TRAIL promotes apoptosis in ovarian cancer cells. Ovarian cancer ascites attenuate TRAIL-induced apoptosis raising the possibility that OPG contained in ascites may abrogate the anti-tumor activity of TRAIL.

**Methods:**

Determination of OPG levels in ascites was measured by ELISA. Effect of OPG on TRAIL-induced cell death was determined by XTT and colony forming assays in ovarian cancer cell lines and primary tumor cells. Apoptosis was assessed by ELISA.

**Results:**

We found that recombinant OPG and malignant ascites attenuates TRAIL-induced cell death and apoptosis in a dose-dependent manner in ovarian cancer cell lines and primary ovarian tumor cells. OPG is present at high levels in the ascites of patients with ovarian cancer. We found a positive correlation between the levels of OPG in ascites and the ability of the ascites to attenuate TRAIL-induced cell death. The anti-apoptotic effect of ascites was not reversed by co-incubation with an OPG blocking antibody.

**Conclusions:**

OPG and malignant ascites protect ovarian cancer cells from TRAIL-induced apoptosis. Although malignant ascites contain high levels of OPG, OPG is not a critical component that contributes to ascites-mediated attenuation of TRAIL-induced apoptosis.

## Introduction

Ovarian cancer (OC) is the fifth cause of cancer-related death in women, the second most common gynecological cancer, and the leading cause of death from gynecological malignancies [1,2]. Over 70% of patients with OC present with late stage disease, with dissemination of tumor implants throughout the peritoneal cavity [1-4]. Only 10-15% of these patients maintain complete response after standard first line treatments, implying that most patients will relapse. Indeed, the five-year survival of patients with late stage disease remains at < 30% with a median survival of 39 months [5]. One of the main obstacles to effective treatment is the failure of initial therapy to eradicate a sufficient number of tumor cells to prevent disease recurrence.

One of the new promising anti-cancer therapies for OC is the tumor necrosis factor-related apoptosis-inducing ligand (TRAIL) [6]. TRAIL is a member of the tumor necrosis factor family and has the ability to selectively induce apoptosis in tumor cells with little toxicity to normal cells. TRAIL has been shown to induce apoptosis in a wide range of tumor cells *in vitro and in vivo*, including OC cells [7-14]. TRAIL triggers apoptosis by interacting with TRAIL death receptors expressed by target cells. TRAIL binds to multiple receptors including TRAIL R1 (DR4), TRAIL R2 (DR5), TRAIL R3 (DcR1), TRAIL R4 (DcR2) [15-18]. Only TRAIL R1 and TRAIL R2 transmit an apoptotic signal. In contrast, TRAIL R3 and TRAIL R4 act as decoy receptors and are incapable of transmitting an apoptotic signal. Soluble TRAIL also binds with low affinity to soluble osteoprotegerin (OPG) [19]. Binding of TRAIL to its death receptors allows the recruitment of Fas-associated protein with death domain (FADD) and pro-caspase 8/10 leading to the formation of the death-inducing signaling complex (DISC). In OC cells, activated caspase-8 cleaves pro-apoptotic Bcl-2 family member Bid to form truncated Bid (tBid), which can then interact with Bax/Bak leading to mitochondrial permeabilization and activation. This interaction increases the release of cytochrome c from the mitochondria resulting in the downstream activation of effector caspases [20].

OPG is a secreted member of the TNF receptor superfamily. It can bind to the receptor activator of NFκB ligand (RANKL) and functions as a soluble decoy receptor for RANKL. OPG regulates the homeostasis of bone remodeling by preventing RANKL from binding to its receptor RANK [21]. OPG also acts as a decoy receptor of TRAIL and neutralizes its function [19]. Several reports have shown that OPG is a survival factor and can block TRAIL-induced apoptosis in tumor cells. For example, human prostate cancer cells were shown to secrete OPG at concentrations sufficient to inhibit TRAIL-induced apoptosis *in vitro*[22,23]. Similarly, multiple myeloma cells were protected from TRAIL-induced apoptosis by OPG produced from osteoblast-like cells and bone marrow stroma cells [24]. OPG produced by breast cancer cells enhances tumor cell survival *in vitro* and *in vivo* by inhibiting TRAIL-induced apoptosis [25-28]. The production of OPG in colorectal cancer cells and the addition of exogenous OPG to colorectal cancer cells both caused resistance to TRAIL-induced apoptosis [29]. The secretion of OPG in the bone microenvironment by either tumor cells or bone marrow stromal cells thus appears to be a critical survival factor for tumor cells. Furthermore, the production and release of OPG into the serum is higher in patients with late stage metastatic colon and prostate cancers suggesting that OPG might exert anti-apoptotic effect during the metastatic process [29,30]. This is further supported by the observation that overexpression of OPG is associated with significantly worse overall survival and relapse-free survival in colon cancer patients [31]. Moreover, overexpression of the OPG protein is an independent risk factor for colon cancer recurrence [31].

Recent data suggest that malignant ascites can affect tumor cell behavior by promoting cell growth, invasion, and survival [32-34]. Specifically, ascites from patients with advanced OC exert a protective effect against TRAIL- and drug-induced apoptosis by inducing survival pathways in tumor cells [32,33,35]. In addition, the presence of high levels of OPG in malignant ascites was recently associated with shorter progression-free survival in patients with OC [36]. These observations raise the possibility that OPG may protect OC cells from TRAIL-induced apoptosis and that OPG production in malignant ascites may be a critical survival factor. In this study, we assessed whether recombinant OPG attenuates TRAIL-induced apoptosis in OC cell lines and primary tumor cells. In addition, we determined whether OPG in ascites facilitates survival of OC cells.

## Methods

### Malignant ascites, primary tumor cells and cell lines

The study was approved by the institutional review board of the Centre Hospitalier Universitaire de Sherbrooke. Informed consent was obtained from women that undergone surgery by the gynecologic oncology service for OC. Malignant ascites were obtained at the time of initial cytoreductive surgery for all patients. All ascites were supplied by the Banque de tissus et de données of the Réseau de Recherche en Cancer of the Fonds de la Recherche en Santé du Québec (FRSQ) affiliated with the Canadian Tumor Repository Network (CTRNet). Malignant ascites were centrifuged at 1000 rpm for 15 min and supernatants were stored at −20°C until assayed for protein content or XTT. Primary tumor cells were isolated as follow: ovarian cancer ascites were centrifuged at 1000 rpm for 15 min and cells were washed twice with OSE medium (Wisent, St-Bruno, Québec, Canada). Cells were then resuspended in OSE medium supplemented with 10% FBS, β-estradiol (10^-8^ M), 2 mM glutamine, antibiotics and fungizone and plated into 75 cm^2^ flasks. All floating cells were removed the next day. Tumor cell samples were used at low passage (< 10). All primary tumor cells were obtained from patients with advanced serous OC. To ensure that these cells were tumor cells, they were stained with epithelial tumor markers anti-CA125 and cytokeratine 8/18 and with fibroblast specific marker fibroblast antigen. As shown in Additional file 1: Figure S1, the human fibroblastic cell line HFL-1 (control) was positive for fibroblast antigen but negative for cytokeratine 8/18 as expected. In contrast, primary tumor cells OVC215A and OVC399A stained positive for cytokeratine 8/18 and CA125 and negative for fibroblast antigen, confirming that most cells are tumor cells. The OC cell lines CaOV3 and OVCAR3 were obtained from American Type Culture Collection, (Manassas, VA) and maintained in a humidified 5% CO_2_ incubator at 37°C. Cells were passaged twice weekly. OVCAR3 cells were maintained in RPMI-1640 (Wisent, St-Bruno, QC, Canada) supplemented with 20% FBS, insulin (10 mg/L), glutamine (2 mM) and antibiotics. CaOV3 cells were cultured in DMEM/F12 (Wisent) supplemented with 10% FBS, 2 mM glutamine and antibiotics.

### Reagents

Recombinant human TRAIL was purchased from PeproTech. (Rocky Hill, NJ). Recombinant human OPG and the anti-OPG neutralizing antibiody was purchased from R&D Systems (Mineapolis, MN). XTT reagent (2,3-bis-(2-methoxy-4-nitro-5-sulfo-phenyl)2H-tetrazolium-5-carboxonilide) was from Life Technology (Burlington, ON). The specific fibroblast antigen antibody was from Oncogen Research Product (clone AS02) (San Diego, CA). Anti-CA125 (M11) was from Dako (Burlington, ON) and anti-cytokeratine 8/18 antibody was from ZYMED laboratories Inc. (San Francisco, CA).

### Cell viability assays

In some experiments cell viability was determined by XTT assay. Briefly, CaOV3 or OVCAR3 cells were plated at 20,000 cells/well in 96-well plates in complete medium. The next day, cells (confluence 60-70%) were exposed to medium containing OPG or ascites (10%) for 1 h after which TRAIL was added to the medium for total of 48 h. At the termination of the experiment, the culture media was removed and a mixture of PBS and fresh media (without phenol red) containing phenazine methosulfate and XTT was added for 30 min at room temperature. The O.D. was determined using a microplate reader at 450 nm (TecanSunrise, Research Triangle Park, NC). The percentage of cell viability was defined as the relative absorbance of TRAIL-treated cells versus untreated (no TRAIL, no ascites, no OPG) cells. Cells were also challenged with TRAIL in fresh medium supplemented with ascites (10%) and. To neutralize OPG in ascites, anti-OPG antibodies (10 μg/ml) were incubated for 1 h with ascites. The OPG-neutralized ascites were then added to cells and they were challenged with TRAIL for 48 h. In other experiments, cells were challenged with increasing concentration of TRAIL (0 to 50 ng/ml) in either fresh medium or medium containing ascites (10%) and the concentration of TRAIL allowing 50% cell viability was defined as the IC_50_. The anti-apoptotic activity of OC ascites was expressed as TRAIL IC_50_ with ascites/IC_50_ without ascites. Protective ascites were defined as those with a ratio ≥ 2.

For clonogenic survival assays, cells were plated into 25 mm^2^ tissue culture plates in standard medium. The next day, cells were incubated for 90 min in medium containing OPG (25 ng/ml) or ascites (10%) and TRAIL (50 ng/ml) was added to the medium for either 3 h or 48 h. Cells were then washed with PBS and incubated in fresh medium into 6-well plates at the different densities for 14 days. Cells were fixed and stained with crystal violet. The number of colonies, consisting of > 50 cells, in triplicate was counted.

### Apoptosis

Cells were incubated in medium containing OPG (25 ng/ml) for 1 h after which TRAIL (50 ng/m) was added to the medium and cells were incubated for 24 h. The release of nucleosomal DNA into the cytoplasm as a measure of apoptosis was determined using the Cell Death Detection ELISA Kit (Roche, Laval, Québec, Canada) according to the manufacturer’s instruction. The absorbance was determined using a microplate reader at 410 nm.

### Determination of OPG concentration by ELISA

OPG levels were determined using an ELISA from E Biosciences (San Diego, CA). The assays were performed in duplicate according to the manufacturer’s protocols. The detection threshold was 4.5 pg/ml for OPG and the intra-assay variability was 4.3-7.9%.

### Statistical analysis

Experiments were performed in triplicate, and data presented as mean ± SD. Student’s paired *t*-test was used to analyze differences between the treatment conditions and their controls. Dose responses were compared by ANOVA test. The threshold for statistical significance is *P* < 0.05.

## Results

### OPG and malignant ascites protects OC cells from TRAIL-induced apoptosis

**Figure 1:**
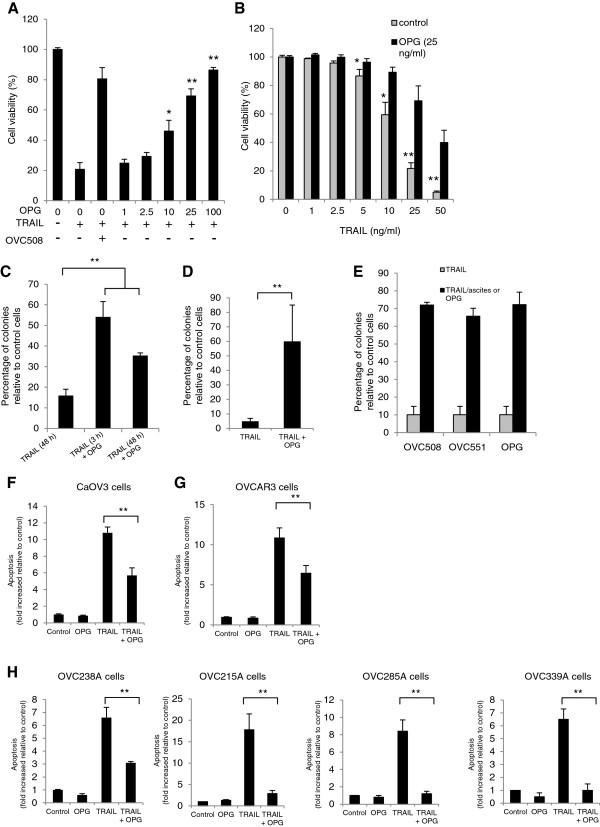
**TRAIL-induced apoptosis can be attenuated by OPG and malignant ascites.** (**A**) CaOV3 cells were challenged with TRAIL (10 ng/ml) and increasing concentrations of recombinant OPG was added to the cultures. Cell viability was assessed after 48 h by XTT assay and was expressed as the percentage relative to control cells. (**B**) CaOV3 cells were challenged with increasing concentrations of TRAIL and a fixed dose of OPG (25 ng/ml for 48 h after which cell viability was assessed. (**C**) CaOV3 cells were challenged with TRAIL (10 ng/ml) and OPG (25 ng/ml) for either 3 h or 48 h. The cells were washed and fresh medium was added. Viable colonies were counted after 14 days and data were expressed as% of colonies in control (untreated) cells. (**D**) OVCAR3 cells were challenged with TRAIL (50 ng/ml) and OPG (25 ng/ml) for 48 h and assessed as above. (**E**) CaOV3 cells were challenged with TRAIL (10 ng/ml) and either OVC508 or OVC551 ascites (10% v/v) or OPG (25 ng/ml) for 48 h and viable colonies were counted after 14 days. CaOV3 cells (**F**), OVCAR3 cells (**G**) and primary tumor cells OVC238A (**H**) were challenged with either OPG (25 to 500 ng/ml), TRAIL (10 to 100 ng/ml) or both and apoptosis was assessed 24 h later. Apoptosis was expressed as fold increased relative to control (untreated) cells. Data are expressed as means of triplicates from three independent experiments ± SD. * *P* < 0.01, ** *P* < 0.001 compared to control.

Because OPG protects prostate and breast cancer cells from TRAIL-induced apoptosis [22-28], we hypothesized that OPG could also attenuate TRAIL-induced apoptosis in OC cells. To this end, we performed dose–response experiments with exposure of OPG at concentrations ranging from 0 to 100 ng/ml for 48 h in TRAIL treated CaOV3 cells. Cell viability was assessed using an XTT assay. As shown in Figure 1A, OPG significantly decreased TRAIL-induced cell death at concentrations > 2.5 ng/ml (*P* < 0.001). Exposure of CaOV3 cells to ascites OVC508, which contain 3570 pg/ml of OPG, also attenuated TRAIL-induced cell death, consistent with previous findings [32,33]. When CaOV3 cells were treated with increasing concentrations of TRAIL (0 to 50 ng/ml), OPG (25 ng/ml) was effective to significantly (*P* < 0.001) attenuate TRAIL-induced cell death (Figure 1B). To extend these findings, CaOV3 cells were challenged with 10 ng/ml of TRAIL for 3 h and 48 h in the presence or absence of OPG (25 ng/ml). Cell viability was assessed by counting the number of viable colonies after two weeks. Exposure to OPG significantly increased the number of colonies when compared to TRAIL-only treated cells (Figure 1C). To ensure that these findings were not limited to a single cell line, a similar experiment was performed with OVCAR3 cells (Figure 1D). Exposure to OPG also significantly increased the number of viable colonies after two weeks. To confirm that ascites attenuate TRAIL-induced cell death, colony forming assays were performed with OVC508 and OVC551 ascites (3570 pg/ml and 19620 pg/ml of OPG respectively) and showed a level of protection from TRAIL-induced cell death similar to OPG (25 ng/ml) (Figure 1E). In agreement with these findings, OPG attenuated TRAIL-induced apoptosis in CaOV3 cells, as measured by oligosomal DNA fragmentation (Figure 1F). Apoptosis was measured in OVCAR3 cells exposed to TRAIL and OPG and consistently showed that OPG attenuated TRAIL-induced apoptosis (Figure 1G). To further extend these observations, primary tumor cells were isolated from the ascites of four patients with advanced OC and OPG protection was consistently seen in these cells (Figure 1H). Taken together, these data suggest that OPG and ascites attenuate TRAIL-induced apoptosis. In addition, they suggest that OPG could be one of the determinants in ascites that confer protection against TRAIL.

**Figure 2:**
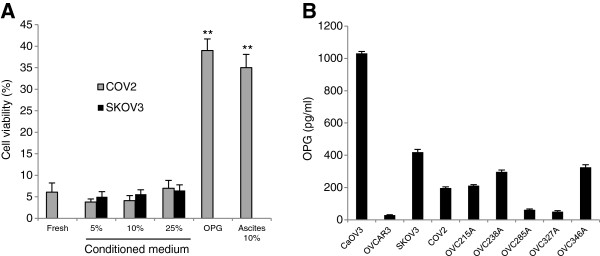
**Conditioned medium from OC cells do not suppress TRAIL-induced cell death.** CaOV3 cells were challenged with TRAIL (50 ng/ml) and either fresh medium (Fresh), 5-25% SKOV3 or COV2 conditioned medium, or OPG (25 ng/ml) or ascites (10%) for 48 h, and cell viability was determined by XTT assay. Data are expressed as means of triplicates from three independent experiments ± SD. ** *P* < 0.001 compared to control. (**B**) OPG concentrations in the cell supernatant of TRAIL sensitive OC cell lines CaOV3 and OVCAR3, TRAIL resistant OC cell lines SKOV3 and COV2, and human primary ovarian tumor cells OVC215A, OVC238A, OVC285A, OVC327A and OVC346A were measured by ELISA after culture of confluent cells for 48 h.

It was previously shown that tumor cells may secrete sufficient levels of OPG in the medium to attenuate TRAIL-induced apoptosis [22,26-28]. Thus, CaOV3 cells were cultured with conditioned media (96 h) from the TRAIL-resistant OC cell line COV2 and SKOV3 in the presence of TRAIL and cell viability was assessed by XTT assay. The hypothesis was that TRAIL-resistant OC cell lines could secrete enough OPG in the medium to confer resistance to TRAIL. However, in contrast to recombinant OPG (25 ng /ml) and ascites (10%), conditioned medium from COV2 and SKOV3 cells (5% to 25%) failed to attenuate TRAIL-induced cell death (Figure 2A). The data suggest that TRAIL-resistant OC cells do not produce enough OPG *in vitro* to suppress TRAIL-induced cell death in CaOV3 cells. An ELISA was used to measure OPG secreted into the medium of OC cell lines, including SKOV3 and COV2 cells, and into the medium of primary ovarian tumor cells when cultured for 48 h (Figure 2B).

### Presence of OPG in malignant ascites from patients with advanced OC

To assess whether OPG could be one of the determinants in ascites that contribute to ascites-mediated inhibition of TRAIL-induced apoptosis, we measured OPG by performing ELISA analysis of the acellular fraction of ascites obtained at the time of the initial surgery from 40 patients with OC (Additional file 2: Table S1). Most (70%) were serous sub-types and had advanced (stage III/IV) disease. ELISA analysis demonstrated that ascites contain OPG at very different levels (median 11.2 nM, range: 0.18-453 nM). This median OPG concentration in OC ascites (11.2 nM) is however lower than the apparent dissociation constant for the binding of OPG to TRAIL that is around 400 nM [19].

**Figure 3:**
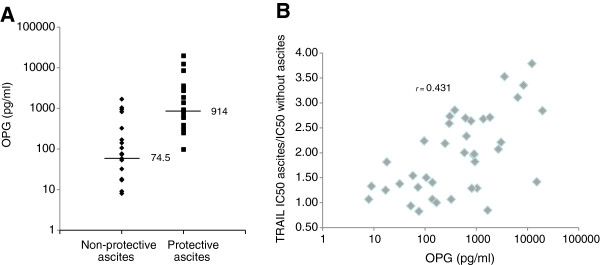
**Concentration of OPG in ascites according to their anti-apoptotic effect against TRAIL.** (**A**) OPG ascites levels in 40 patients with OC in non-protective versus protective ascites against TRAIL as defined in Methods. Horizontal bars represent median values. (**B**) Correlation chart of OPG levels in ascites versus the anti-apoptotic activity of ascites. The correlation coefficient (*r*) is indicated.

To determine whether there is a correlation between OPG levels in ascites and their ability to attenuate TRAIL-induced apoptosis, TRAIL IC_50_ was determined from cell viability curves done with the CaOV3 cell line. The anti-apoptotic activity of OC ascites was expressed as TRAIL IC_50_ with ascites/IC_50_ without ascites. Protective ascites were defined as those with a ratio ≥ 2. The protein concentration was not significantly different in protective and non-protective ascites (data not shown). As shown in Figure 3A, the median level of OPG was significantly higher in ascites with anti-apoptotic activity as compared to those without (*P* < 0.001). Furthermore, there was a positive correlation between OPG levels in ascites and their anti-apoptotic activity against TRAIL with correlation coefficient (*r*) of 0.431 (Figure 3B). These data support the hypothesis that OPG is one of the factors that promote ascites-mediated attenuation of TRAIL-induced apoptosis.

### Ascites-mediated attenuation of TRAIL-induced apoptosis is OPG-independent

**Figure 4:**
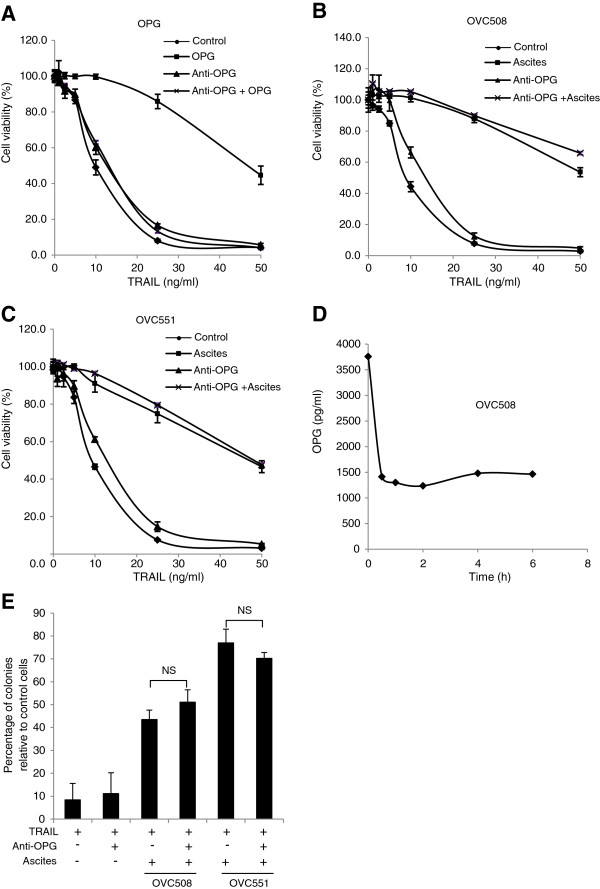
**The anti-apoptotic effect of ascites cannot be reversed by OPG neutralizing antibodies.** (**A**) CaOV3 cells were challenged with increasing concentrations of TRAIL and OPG (25 ng/ml) in the presence or absence of anti-OPG antibodies (10 μg/ml) for 48 h. Cell viability was determined by XTT assay and expressed as the percentage relative to control (untreated) cells. CaOV3 cells were challenged with TRAIL, OVC508 ascites (10%) (**B**) or OVC551 ascites (10%) (**C**), in the presence or absence of anti-OPG antibodies (10 μg/ml) for 48 h. Cell viability was assessed as above. (**D**) OVC508 ascites was incubated with anti-OPG antibodies (10 μg/ml) for up to 6 h. Protein G agarose was then added for 1 h and centrifuged and OPG levels were determined in the supernatant by ELISA. (**E**) CaOV3 cells were challenged with TRAIL (10 ng/ml), ascites (10%) and anti-OPG (10 μg/ml) for 48 h. The cells were washed and fresh medium was added. Viable colonies were counted after 14 days and data were expressed as% of colonies in control (untreated) cells. Data are expressed as means of triplicates from three independent experiments ± SD. NS indicates that differences did not reach statistical significance with *P* > 0.05.

Although malignant ascites and OPG both attenuate TRAIL-induced apoptosis, it is unclear whether OPG contributes to the inhibition of TRAIL-induced apoptosis by ascites. We thus used a blocking antibody to neutralize OPG in ascites. CaOV3 cells were untreated (control), treated with TRAIL alone or with a combination of TRAIL with OPG (25 ng/ml) in the presence or absence of an OPG blocking antibody, and cell viability was assessed by XTT. As expected, the addition of the OPG blocking antibody completely abrogated the protective effect of OPG against TRAIL (Figure 4A). In contrast, the addition of the OPG blocking antibody had no effect on the anti-apoptotic activity of OVC508 (Figure 4B) and OVC551 (Figure 4C) ascites despite the fact that 62-66% of the OPG in ascites was neutralized (Figure 4D). To confirm these data, we performed colony forming assays. In agreement with the XTT assays, although exposure to OVC508 and OVC551 ascites significantly inhibited TRAIL-induced cytotoxicity in CaOV3 cells, the addition of the anti-OPG antibody failed to alter the anti-apoptotic effect of OVC508 and OVC551 ascites (Figure 4E). Altogether, the results suggest that OPG is not a critical component that contributes to ascites-mediated attenuation of TRAIL-induced apoptosis.

## Discussion

In this study, we tested the hypothesis that the anti-apoptotic effect of malignant ascites against TRAIL could be related to the presence of high levels of OPG. Previous studies have established that OPG acts as a survival factor, at least *in vitro*, by attenuating TRAIL-induced apoptosis in colon, breast and prostate cancer cells [22-29]. In addition, OPG appears to be a critical component of the bone microenvironment. Our *in vitro* results demonstrated that recombinant OPG protected CaOV3 and OVCAR3 cell lines from TRAIL-induced apoptosis in a dose-dependent manner. Furthermore, tumor cells isolated from malignant ascites were also protected from TRAIL-induced apoptosis by the addition of OPG, indicating that OPG acts as a survival factor for OC cells. Minimal concentrations of OPG required to significantly inhibit TRAIL-induced apoptosis in OC cell lines were in the order of 10 ng/ml. The level of OPG present in malignant ascites varied considerably between ascites and in many instances was too low (< 1 ng/ml) to counteract the apoptotic action of TRAIL. Nonetheless, we found a positive correlation between the level of OPG present in ascites and the ability of ascites to attenuate TRAIL-induced apoptosis. These observations may support the proposition that OPG, as an extracellular factor in malignant ascites, may function as a survival factor for OC cells.

The anti-apoptotic effect of recombinant OPG could be completely reversed by cotreatment with an anti-OPG blocking antibody whereas the antibody itself had no effect on apoptosis levels in OC cells (data not shown). CaOV3 cells treated with TRAIL in the presence of OVC508 and OVC551 ascites containing 3.6 ng/ml and 19,6 ng/ml respectively of OPG showed lower level of cell death compared to those treated with TRAIL alone. Although this anti-apoptotic effect of ascites was hypothesized to be due, at least in part, to the presence of OPG, incubation of CaOV3 cells with the OPG blocking antibody had no effect on the anti-apoptotic property of OVC508 and OVC551, suggesting that OPG is not a critical survival factor in ascites.

The source of OPG in malignant ascites remains to be established but it has been shown that a number of different cell types are capable of producing OPG. Data from other studies suggest that prostate and breast cancer cells can produce OPG [22,23,25-28]. Our data demonstrated that OC cancer cell lines and primary tumor cells isolated from ascites also produce OPG with concentration ranging from 30 to 1031 pg/ml. These concentrations were well within the range of those found in malignant ascites. The overall contribution of the various cell populations to the total OPG levels in ascites will depend on the number of tumor cells in relation to other cells. Stromal cells have been shown to be a significant source of OPG [25,37]. In this context, it could be speculated that, in addition to tumor cells, human peritoneal mesothelial cells, which are an important constituent of malignant ascites, could also be a source of OPG.

In summary, although most malignant ascites have measurable OPG level, these concentrations do not reach the level required to attenuate TRAIL-induced apoptosis in the majority of ascites. In addition, blockade of endogenous OPG in ascites do not affect their anti-apoptotic activity. Thus, the presence of OPG in malignant ascites by itself is an unlikely mechanism by which ascites attenuate TRAIL-induced apoptosis.

## Competing interests

The authors report no conflict of interest.

## Authors’ contributions

DL participated in the design of the study, performed all the experiments and generated all the data. IM was responsible for obtaining the ascites and the clinical data. CR participated in the design of the study and helped to draft the manuscript. AP conceived the study, participated in its design and drafted the manuscript. All authors read and approved the final manuscript.

## Supplementary Material

Additional file 1 **Figure S1.** Human fibroblast 1 cell line. Human primary ovarian cancer cells. Click here for file

Additional file 2 **Table S1.** Histopathologic data of OC ascites. Click here for file
