# Supplementary material for: Osteoprotegerin (OPG) protects ovarian cancer cells from TRAIL-induced apoptosis but does not contribute to malignant ascites-mediated attenuation of TRAIL-induced apoptosis
Source: J Ovarian Res. 2012 Nov 15;5:34. doi: 10.1186/1757-2215-5-34 (PMC3507713; doi:10.1186/1757-2215-5-34)
Supplement: Additional file 1 — Figure S1. Human fibroblast 1 cell line. Human primary ovarian cancer cells. [file 1757-2215-5-34-S1.ppt]

## Slide 1
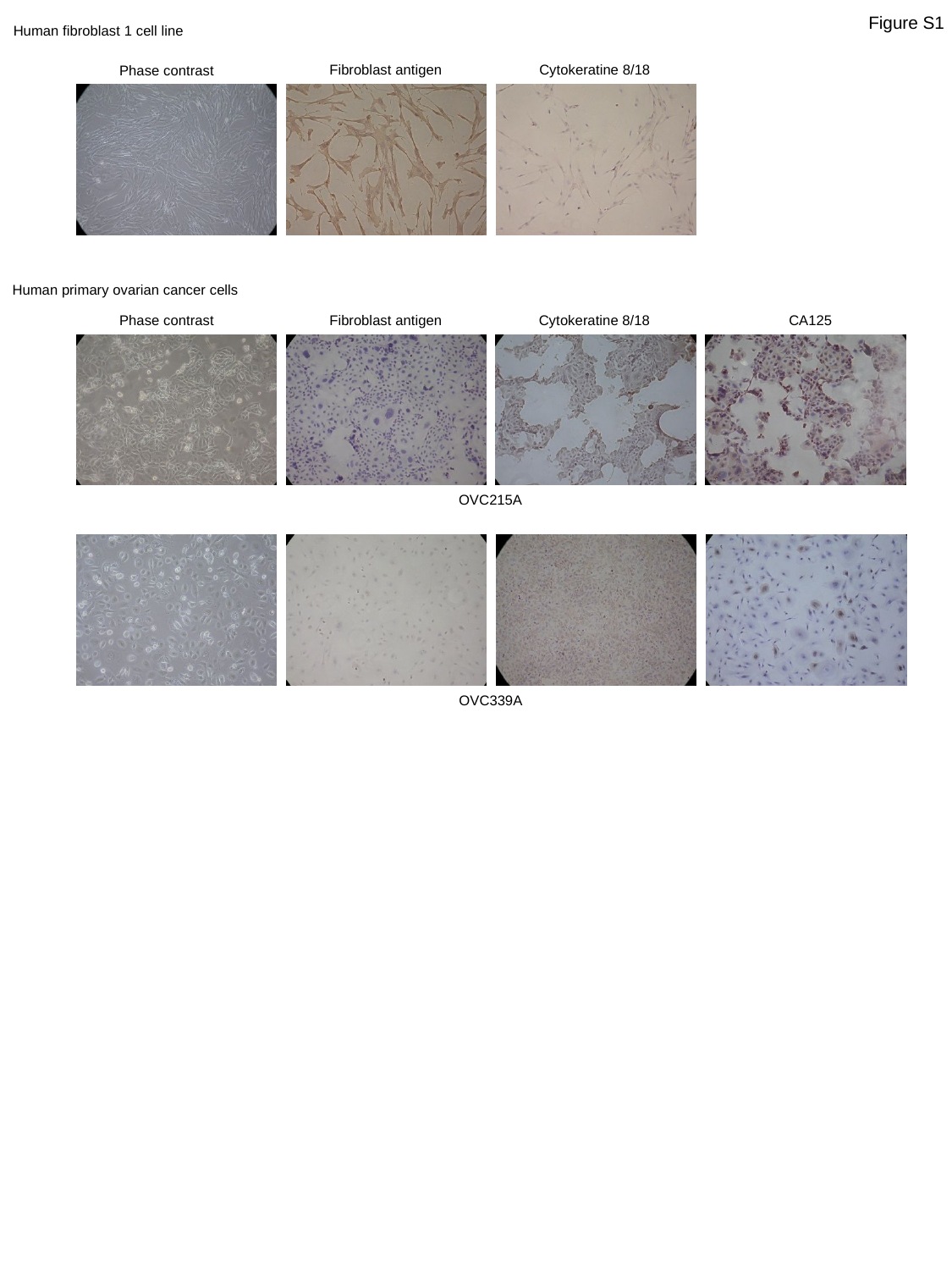

Figure S1
Human fibroblast 1 cell line
Fibroblast antigen
Cytokeratine 8/18
Phase contrast
Human primary ovarian cancer cells
Cytokeratine 8/18
CA125
Phase contrast
Fibroblast antigen
OVC215A
OVC339A

## Slide 2
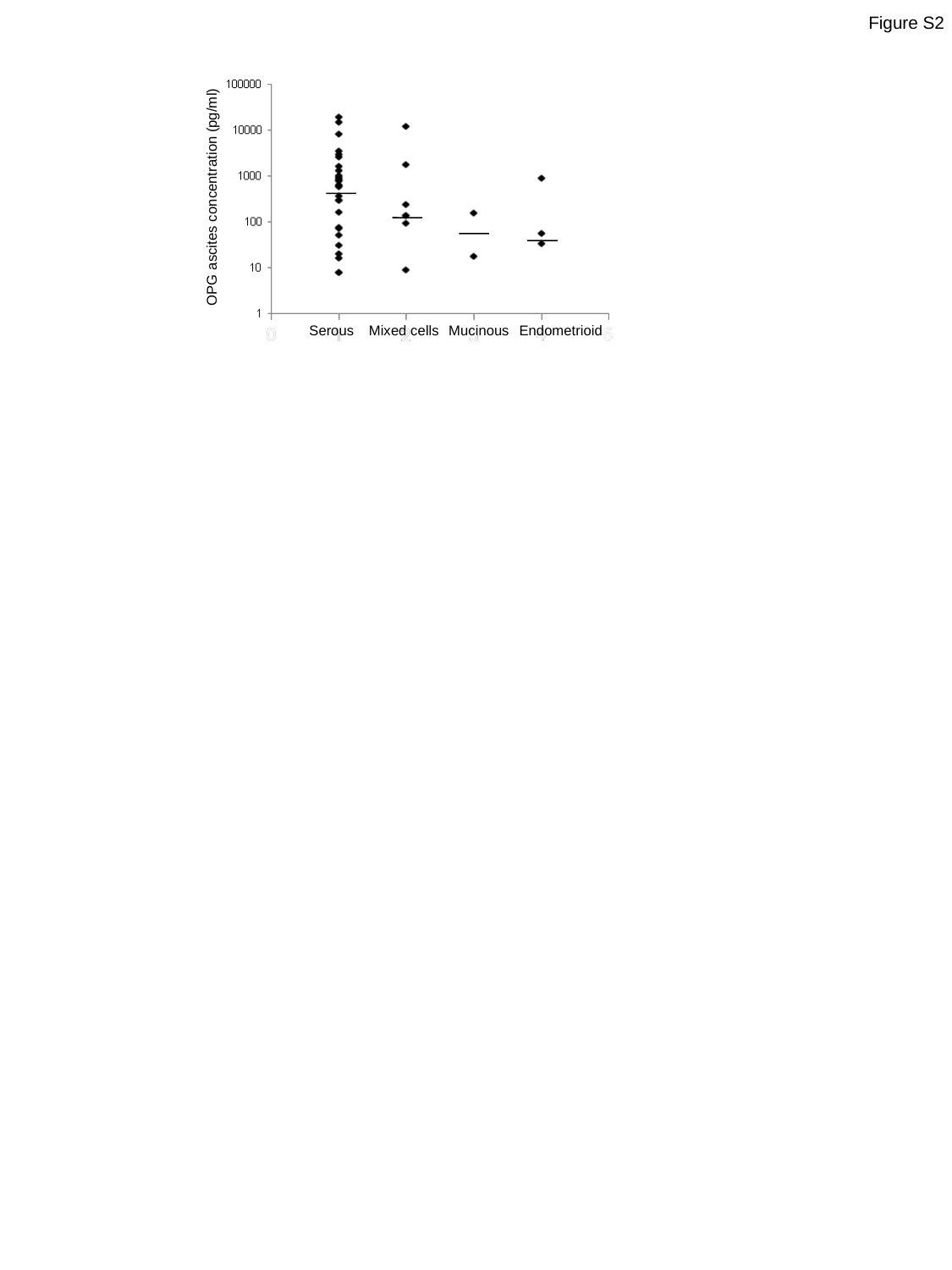

Figure S2
OPG ascites concentration (pg/ml)
Serous
Mixed cells
Mucinous
Endometrioid
