# Supplementary material for: Osteoprotegerin (OPG) protects ovarian cancer cells from TRAIL-induced apoptosis but does not contribute to malignant ascites-mediated attenuation of TRAIL-induced apoptosis
Source: J Ovarian Res. 2012 Nov 15;5:34. doi: 10.1186/1757-2215-5-34 (PMC3507713; doi:10.1186/1757-2215-5-34)
Supplement: Additional file 2 — Table S1. Histopathologic data of OC ascites. [file 1757-2215-5-34-S2.docx]

Table 1S. Histopathologic data of OC ascites

| Ascites  (n=40) | Histopathology | Grade | Stage | Prior  chemotherapy |
| --- | --- | --- | --- | --- |
| COV2 | Serous | 3 | IV | No |
| COV10 | Serous | 3 | IV | No |
| OVC40 | Serous | 3 | IIIC | No |
| OVC41 | Serous | 3 | IV | N/A |
| OVC48 | Serous | 3 | N/A | No |
| OVC346 | Serous | 3 | IIIC | No |
| OVC350 | Serous | 3 | IIIC | Yes |
| OVC361 | Serous | 2 | IIIC | No |
| OVC374 | Mucinous | N/A | IC | Yes |
| OVC380 | Mucinous | N/A | IA | No |
| OVC395 | Serous | 2 | IV | No |
| OVC405 | Serous | 2 | IIIC | No |
| OVC409 | Mixed cell | 3 | IIC | No |
| OVC410 | Endometrioid | 2 | IIIA | No |
| OVC414 | Endometrioid | 1 | IV | No |
| OVC427 | Serous | 2 | IIIC | No |
| OVC432 | Mixed cell | 3 | IC | No |
| OVC439 | Serous | 3 | IIIC | No |
| OVC448 | Mixed cell | 3 | IIIB | Yes |
| OVC451 | Mixed cell | 3 | IC | No |
| OVC461 | Serous | 1 | IA | No |
| OVC463 | Serous | 2 | IIIC | Yes |
| OVC469 | Serous | 1 | IIIC | No |
| OVC472 | Mixed cell | 3 | IIC | No |
| OVC483 | Serous | N/A | IA | No |
| OVC488 | Serous | 3 | IIIC | No |
| OVC489 | Endometrioid | 2 | IIIB | No |
| OVC500 | Mixed cell | 1 | IA | No |
| OVC503 | Serous | N/A | IV | Yes |
| OVC508 | Serous | 3 | IV | No |
| OVC509 | Serous | 2 | IV | No |
| OVC517 | Serous | 2 | IV | No |
| OVC523 | Serous | 3 | IIIC | No |
| OVC530 | Mixed cell | 2 | IIC | No |
| OVC535 | Serous | 2 | IV | No |
| OVC547 | Serous | 3 | IV | No |
| OVC551 | Serous | 3 | IIIC | No |
| OVC552 | Serous | 3 | IIC | No |
| OVC563 | Serous | 3 | IIIC | No |
| OVC572 | Serous | 1 | IB | No |
